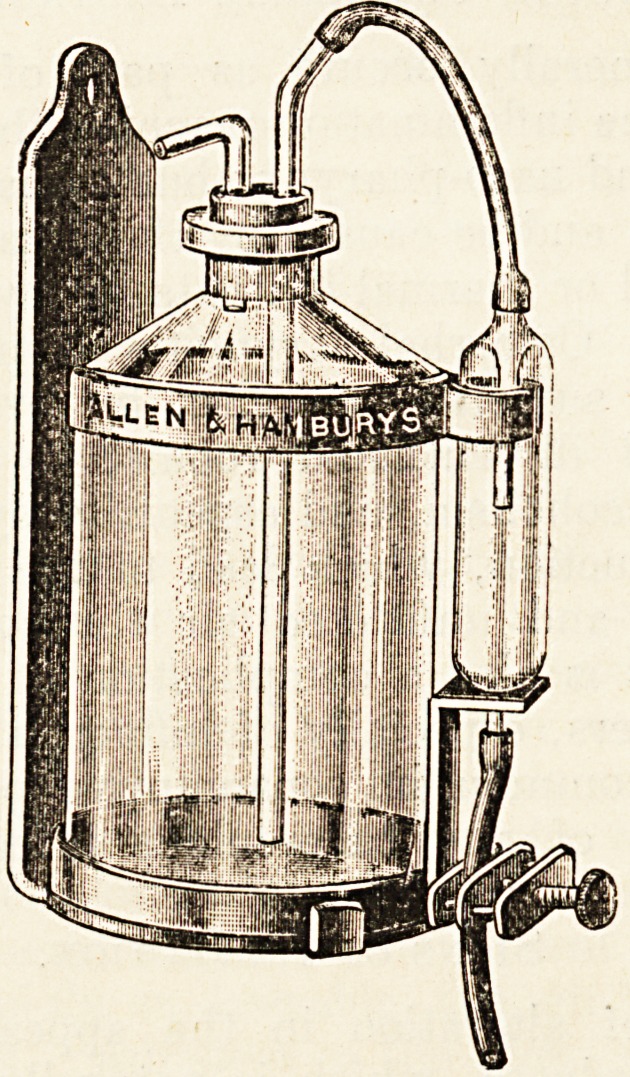# Continuous Irrigation with Hydrogen Peroxide as a Treatment for Septic Sinuses, Etc.

**Published:** 1910-01-29

**Authors:** R. Douglas Laurie

**Affiliations:** House Surgeon, Royal Hants County Hospital, Winchéster


### RESIDENT MEDICAL OFFICERS' DEPARTMENT.

### CONTINUOUS IRRIGATION WITH HYDROGEN PEROXIDE AS A TREATMENT FOR SEPTIC SINUSES, Etc.

By R. DOUGLAS LAURIE, M.B.; House Surgeon, Royal Hants County Hospital, Winchester.

As an application to septic sinuses and cavities hydrogen peroxide is in everyday use, and has many advantages over the ordinary carbolic, biniodide, and perchloride lotions. No toxic effects arise from its use as may occur with these latter drugs.

The oxygen liberated from the hydrogen peroxide by contact with the necrosed tissue is a powerful germicide and stimulant to the walls of the cavity. It also acts mechanically in carrying effete matter from the surfaces, while its solvent action on blood aids the removal of clots. Its action, however, ceases shortly after application, *i.e.* when all the oxygen is evolved. To get a more prolonged action an apparatus is employed which delivers the peroxide in a slow, steady stream into the cavity. This (see fig.) comprises a reservoir, a tube to indicate the rate of flow, and a clamp to regulate it. The apparatus is placed some two or three feet above the level of the patient, and the delivery tube leads the peroxide to the foot of the sinus or cavity to which it is being applied. It has been found most suitable to use a dilute "5-volumes" solution. With the solution running at the rate of ten to twenty drops per minute six ounces should be sufficient to irrigate the average sinus for four hours; say two hours in the morning and two in the afternoon. It is important that the tube should lie at the deepest part of the sinus or abscess cavity, to ensure the whole surface coming in contact with the peroxide. The mouth of the wound may, with advantage, be kept open by a short piece of wide drainage tube and covered with some absorbent dressing such as gauze or cellulose. The tube may be fixed in position by adhesive strapping.

This treatment has been used in some thirty cases, chiefly appendix abscesses; but also in empyema, tuberculous sinuses in soft tissues and in bones, chronic mastoiditis, etc. The results were very satisfactory, particularly in cases of appendix abscess. In several cases after two or three applications the abscess cavity has become clean, ceased to discharge, and healed in a week or ten days. In a case of large parotid abscess, which contained a quantity of foul-smelling pus—due to B. coli—ordinary drainage and irrigation were employed through two incisions for several days with slight improvement. Continuous irrigation with peroxide was then employed, and in five days only dry dressings were needed. One or two cases have been disappointing, for although at first there was rapid improvement the sinuses refused to heal. In these the peroxide was discontinued after a week and dry gauze plugs inserted; healing then gradually took place. Septic superficial wounds have also shown rapid improvement under continuous peroxide irrigation. If used economically the expense of hydrogen peroxide is not great, and is justified by the more rapid recovery.

## Figures and Tables

**Figure f1:**